# Determination of Glyphosate in Dried Wheat by ^1^H-NMR Spectroscopy

**DOI:** 10.3390/molecules25071546

**Published:** 2020-03-28

**Authors:** Elena Shumilina, Christian Andreasen, Zahra Bitarafan, Alexander Dikiy

**Affiliations:** 1 Department of Biotechnology and Food Science, Norwegian University of Science and Technology, 7491 Trondheim, Norway; alex.dikiy@ntnu.no; 2 Department of Plant and Environmental Science, University of Copenhagen, DK2630 Taastrup, Denmark; can@plen.ku.dk (C.A.); zab@plen.ku.dk (Z.B.)

**Keywords:** crop desiccant, glyphosate, herbicide, NMR, preharvest spraying, Roundup, wheat.

## Abstract

A wheat field was sprayed with a dosage of 1.1 kg a.i./ha Roundup PowerMax 10 days before harvest. The ^1^H Nuclear Magnetic Resonance (NMR) spectroscopy was used for the detection and quantification of the glyphosate (GLYP) in dried wheat spikelets, leaves, and stems. The quantification was done by the integration of the CH_2_-P groups doublet at 3.00 ppm with good linearity. The GLYP content varied between different samples and parts of the plant. On average, the largest content of herbicide was found in leaves (20.0 mg/kg), followed by stems (6.4 mg/kg) and spikelets (6.3 mg/kg). Our study shows that the ^1^H-NMR spectroscopy can be a rapid and reliable tool for GLYP detection and quantification in the field studies.

## 1. Introduction

One of the key challenges in agriculture is weeds, as they compete for water, light, and nutrition with the crop and thus reduce crop production. To tackle this challenge, a variety of herbicides have been developed, however several of them have been banned afterwards due to their health and environmental hazards. Nevertheless, the Transparency Market Research reports foresee an increase of the global herbicides market from 17.37 in 2016 to 29.30 milliards US $ by the end of 2025 [1]. Glyphosate (GLYP) is a nonselective, postemergence herbicide whose market is anticipated to rise at a CAGR of 6.8% from 2017 to 2024 [2].

GLYP-based pesticides are typically applied before the crops are sown and as a preharvest crop-desiccating treatment, to accelerate and homogenize the ripening process. This foliage-applied herbicide acts as a transition-state inhibitor of 5-enolpyruvate shikimate-3-phosphate synthase, interrupting this pathway and leading to a shortage of aromatic amino acids and eventually to the death of the plant [3]. 

GLYP formulations such as Roundup have been approved by regulatory bodies worldwide. However, there have been a number of concerns about their effects on humans, animals, and the environment [4,5]. The review of the German Federal Institute for Risk Assessment concluded in 2013 that the available data were contradictory and far from being convincing with regard to correlations between exposure to GLYP formulations and risk of various cancers [6]. Schinasi and Leon (2014) reported an increased risk of non-Hodgkin lymphoma cancer in workers exposed to GLYP formulations [7]. The World Health Organization International Agency for Research on Cancer classified GLYP as probably carcinogenic in humans [8]. However, in 2015, the European Food Safety Authority reported that GLYP is unlikely to be genotoxic or to pose a carcinogenic threat to humans. Later, they clarified that while carcinogenic GLYP-containing formulations may exist, studies that look solely at the active substance GLYP do not show this effect [9,10]. The WHO and FAO Joint committee on pesticide residues issued a report in 2016 stating that the use of GLYP formulations does not necessarily constitute a health risk, and gave admissible daily maximum intake limits (one mg/kg of body weight per day) for chronic toxicity [11].

GLYP is commonly used in the form of an isopropylamine or trimethylsulfonium salt of GLYP anion. Depending upon the pH, the phosphonic and carboxylic moieties of GLYP might be ionized as well as its amino group can be protonated. Therefore, GLYP may coordinate different numbers of isopropylamine or other cations for one GLYP anion [12]. Commercial GLYP formulations usually contain a monovalent salt of GLYP due to higher water solubility (for example, Roundup) [13].

After application, GLYP is readily transported from the leaves through the plant within the first hours [3,14,15]. The median half-life of GLYP in water varies from a few to 91 days. The main product of GLYP degradation is aminomethylphosphonic acid. A comprehensive review on GLYP uptake and persistence was published by the International Program on Chemical Safety [16].

Monitoring herbicide turnover in human, water, soil, and plants is of crucial importance, due to the toxicity of GLYP. The main analytical methods used to quantify GLYP include thin-layer chromatography, colorimetry, gas chromatography, and high-performance liquid chromatography [16]. These procedures are often laborious, complex, and costly [16]. ^31^P Nuclear Magnetic Resonance (NMR) spectroscopy was successfully used for the monitoring *in vivo* the glyphosate resistance mechanism of plants [3,17]. Other studies report the direct assessment of GLYP levels in human serum and urine without any separation and/or derivatization step [18,19]. Method validation showed that NMR spectroscopy allows for GLYP quantification by the integration of CH_2_–(P) resonances with a good linearity. The relative peak area was a linear function of the glyphosate concentration, with a good significant correlation coefficient (*r* = 0.998) [18,19]. It was concluded that NMR is a rapid (10–20 min) and reliable tool in the detection and quantification of glyphosate in complex fluids [19]. The possibility of NMR peaks overlapping was mentioned as a method limitation.

In this study, ^1^H-NMR spectroscopy was used to quantify GLYP in wheat spikelets, leaves, and stems, treated with the Roundup PowerMax. The proposed protocol is a direct and fast alternative to conventional methods and can be used to monitor GLYP level in real field studies. Our hypothesis was that i) wheat plants sprayed ten days before harvest would still contain glyphosate at harvest, but the concentration would vary between the plant parts and ii) the plant drying will not degrade GLYP.

## 2. Results and Discussion

### 2.1. ^1^H-NMR Spectra of Spikelets, Stems and Leaves Water Extracts

**Figure 1:**
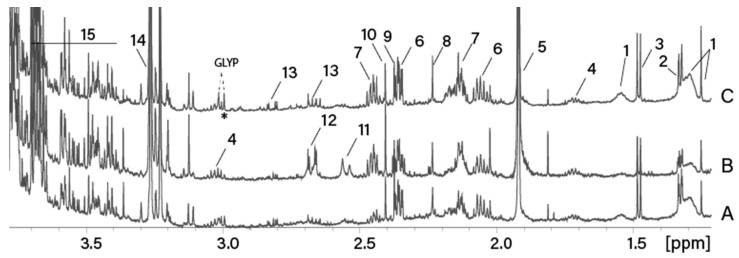
The 1.2–4 ppm region of the ^1^H Nuclear Magnetic Resonance (^1^H-NMR) 600 MHz spectra of the water extracts of wheat leaves (**A**), stems (**B**), and spikelets (**C**), in water at 300K. GLYP—glyphosate,1—fatty acid, 2—threonine; 3—alanine; 4—lysine; 5—acetate; 6—glutamate; 7—glutamine; 8—acetone; 9—pyruvate; 10—succinate; 11—citrate; 12—malate; 13—aspartate; 14—betaine; and 15—sugars (including glucose and mannitol).

The 1.2–4 ppm region of the ^1^H-NMR spectra of the water extracts of GLYP-treated wheat spikelets, stems, and leaves are shown in the Figure 1.

The NMR spectra of all water extracts contain resonances of amino (threonine, alanine, lysine, glutamate, glutamine, and aspartate) and organic (acetic, pyruvic, and succinic) acids, betaine, sugars, and other minor metabolites. All water extracts contain singlet at 1.25 ppm and broad signals at 1.3, 1.55 ppm due to the presence of water-soluble fatty acid. Stems extracts additionally contain citric and malic acids.

Glyphosate resonances were detected in NMR spectra of wheat treated with GLYP. In accordance with Cartigny et. al. [18,19], GLYP has two groups of signals: doublet at 3.00 ppm (*J* = 11.7 Hz) assigned to the CH_2_-P group and a singlet at 3.73 ppm assigned to protons of CH_2_-N group. Singlet at 3.73 ppm is overlapped with the resonances of glucose and other sugars. Doublet at 3.00 ppm can be easily detected in the NMR spectrum of spikelets (Figure 1C). However, spectra of stems and leaves extracts contain resonances of other metabolites (e.g., lysine, GABA) in the region of interest and one of the peaks of the GLYP doublet is overlapped with them. Two methyl groups of isopropylammonium have a doublet at 1.25 ppm, however this signal is severely overlapped with the signal of the water-soluble fatty acid in all spectra.

### 2.2. Linearity Test and Quantification Limits

**Figure 2:**
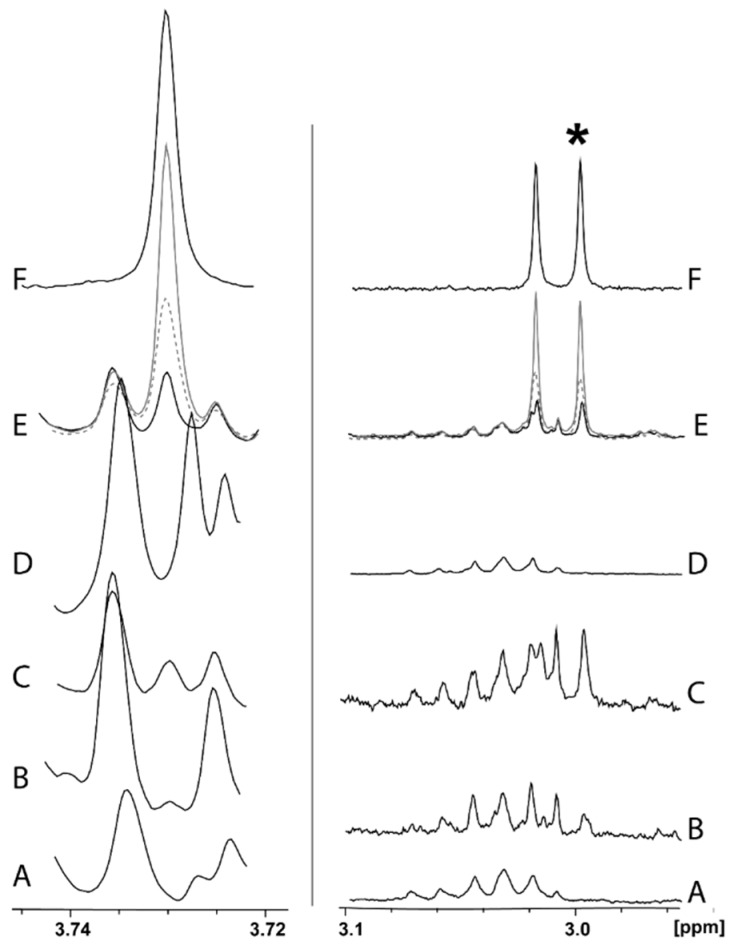
^1^H-NMR spectra (normalized to samples’ weight) of stems (A) and spikelets (D) nontreated with GLYP (data from our laboratory) and GLYP-treated stems (B), leaves (C), spikelets (E), and GLYP (F) standard in water at 300K. E-line shows a reference spectrum of spikelet extract (solid black line) and the resulting spectra after addition of the 10 and 20 µL of GLYP stock solution (gray dashed and solid lines) to the reference extract. Star shows non-overlapped peak that was used for GLYP quantification.

An increasing amount of GLYP standard solution was added to one of the spikelets’ extracts (Figure 2E). Both singlet at 3.73 ppm and one of the peaks in the doublet at 3.00 ppm were used for in the linearity test. The relative peak area of singlet at 3.73 ppm and one of the peaks in the doublet at 3.00 ppm was found to be a linear function of GLYP concentration (0.07 to 0.3 mmol/L) with an acceptable significant correlation coefficients *r* = 0.999 and 0.995, respectively. However, singlet at 3.73 ppm cannot be used for the GLYP quantification because it is overlapped by the sugar’s resonances in leaves and stems extracts. At the same time, one of the doublets peaks at 3.00 ppm is well separated in all spikelets extracts. However, the quantification of GLYP in leaves and stems sometimes was challenging due to the presence of other overlapping signals (Figure 2B,C).

Three NMR samples were prepared from the same spikelet extract for the estimation of the quantification uncertainty that was found to be 1.7%. The concentration was defined as at the limit-of-quantification (LOQ) when the Signal-to-Noise (S/N) was 10:1. With the current experimental design, it was found that the LOQ for GLYP in water extract was about 0.004 mmol/L (S/N = 11). However, this limit might be decreased by increasing of the numbers of scans (NS) during NMR data acquisition.

### 2.3. Glyphosate Quantification

GLYP was quantified by integrating the peak at 3.00 ppm. An average of the three integrations of one of the doublet peaks at 3.00 ppm (Figure 2, peak with the star) multiplied by 2 was used for the quantification. Different concentrations of GLYP were detected in wheat spikelets, leaves, and stems, with an average value of 6.34 (min 3.7 ± 0.1; max 10.4 ± 0.5), 20.02 (min 10.8 ± 0.1; max 28.5 ± 1.5), and 6.4 (min 3.7 ± 0.7; max 14.5 ± 0.2) mg/kg of dry plant, respectively. The obtained results are comparable to literature data on GLYP uptake and dissipation in plants [20]. For example, Roy et. al. performed a field study on the uptake and persistence of GLYP in ripe berries of red raspberries and wild blueberries [21]. Roundup was applied to the field at a higher rate than in our experiment (2 kg a.i./ha, compared with 1.1 kg a.i./ha in our experiment). The residue level of GLYP in fresh berries of blueberries and red raspberries was found to be 3.7 and 5.6 mg/kg, respectively, on the 13th day after application [21]. It needs to be emphasized that the concentration of GLYP was determined in wet berries, while in our experiment all parts of the plant were dried. However, the average amount of GLYP in wheat after 10 days of relatively dry weather is comparable with the results from Roy (6 mg/kg of dry spikes) [21].

## 3. Materials and Methods

### 3.1. Chemicals

Glyphosate standard (certified reference material, TraceCERT^®^) was from Sigma-Aldrich; deuterium oxide (D_2_O, 99.9%) was purchased from Cambridge Isotope Laboratories Inc. (Andover, MA, USA). 3-(trimethylsilyl)-propionic-2,2,3,3-d4 acid sodium salt (TSP, 98 atom % D) was obtained from Armar Chemicals (Dottingen, Switzerland).

### 3.2. Experimental Design

A winter wheat variety (*Triticum aestivum* L.) grown on a field at the research station of University of Copenhagen, Taastrup (55°38’ N, 12°17´ E), Denmark was sprayed with Roundup PowerMax (registered by Monsanto Crop Sciences Danmark A/S) at a dosage of 1.1 kg a.i./ha on the 6th of August 2017. The wheat plants were ripening and had almost hard kernels. Twelve wheat samples were collected randomly after ten days (16th of August) just before the whole field was harvested. Starting 10 m inside the field, samples were taken every ten meters across the field. During the period of application, precipitation was mostly absent, and the average temperature was 16.9 °C. However, it was raining three days before sampling. Wheat plants were divided into spikes, leaves, and stems; dried in a greenhouse; and afterwards sent for analysis to the Norwegian University of Science and Technology. Plants were stored at −40 °C before the NMR analysis.

### 3.3. Polar Metabolite Extraction

Five grams of spikelets, 2 g of leaves, and 2.5 g of stems from each replica were separately extracted into 25 mL of deionized H_2_O. The extraction was carried out for 30 min at room temperature with gentle rotation. Further, water extracts were centrifuged for 10 min at 6500 g. Supernatants were collected and immediately used for the preparation of NMR samples.

### 3.4. NMR Sample Preparation

540 µL of extract were mixed with 60 µL of 10 mM TSP in 20 mM sodium phosphate buffer, pH 7 in D_2_O. The samples were centrifuged (20,000 *g* × 5 min at 20 °C). 530 µL of the supernatant were transferred to a standard 5 mm NMR tube.

### 3.5. NMR Data Acquisition and Processing

^1^H-NMR spectra of the water extracts were acquired at 300 K with a Bruker Avance 600-MHz spectrometer equipped with 5-mm z-gradient TXI (H/C/N) cryoprobe for a total of 40 spectra. The NMR presaturation experiments for water suppression were acquired with the Bruker pulse sequences *noesygppr1d* with the following setting: TD = 64K; relaxation delay (d1) = 4s; SW = 20ppm; NS = 128; RG = 11.3. The spectra were calibrated against TSP standard assigning a chemical shift of 0 ppm to the TSP signal both in ^1^H and ^13^C dimensions. Resonance signals were assigned based on the 2D NMR experiments (^1^H-^1^H TOCSY; JRES, ^1^H-^13^C HSQC, ^1^H-^13^C HMBC), using our previous data and published reference standards (Biological Magnetic Resonance Data Bank and Human Metabolome Database) [21,22,23].

### 3.6. Glyphosate Standard Addition

1 mM glyphosate stock solution was prepared by dissolving the glyphosate powder in 20 mM phosphate buffer, pH 7.0. Two 10 µL aliquots were successively added to the reference (Ref_0; Ref_10; Ref_20) wheat spikelets extract. ^1^H-NMR spectra of Ref_0; Ref_10 and Ref_20 were acquired and processed as described in *NMR Data Acquisition and Processing*. Average integral out of three resonances integration was used for the glyphosate quantification.

## 4. Conclusions

Our results show that ^1^H-NMR spectroscopy can be used for the rapid GLYP detection in the wheat crops. The whole procedure from the extract preparation and data acquisition takes about one hour and does not need any pretreatment. The proposed protocol allowed the detection of the GLYP in dried wheat spikelets, leaves, and stems, after 10 days of the herbicide application. The GLYP quantification in leaves and stems might sometimes be challenging due to the signal overlapping that should be mentioned as a method limitation.
